# Virtual reality assessment of reaching accuracy in patients with recent cerebellar stroke

**DOI:** 10.1186/s44247-024-00107-7

**Published:** 2024-08-12

**Authors:** Khai Du, Leonardo R. Benavides, Emily L. Isenstein, Duje Tadin, Ania C. Busza

**Affiliations:** 1 Department of Neurology, University of Rochester Medical Center, Rochester, NY USA; 2 Department of Neuroscience, University of Rochester Medical Center, Rochester, NY USA; 3 Department of Brain and Cognitive Sciences, University of Rochester, Rochester, NY USA; 4 Center for Visual Science, University of Rochester, Rochester, NY USA; 5 Department of Ophthalmology, University of Rochester Medical Center, Rochester, NY USA; 6 Department of Physical Medicine and Rehabilitation, University of Rochester Medical Center, Rochester, NY USA

**Keywords:** Virtual Reality, Reaching Accuracy, Proprioception, Dysmetria, Cerebellar Stroke, Rehabilitation

## Abstract

**Background:**

Dysmetria, the inability to accurately estimate distance in motor tasks, is a characteristic clinical feature of cerebellar injury. Even though subjective dysmetria can be quickly detected during the neurological examination with the finger-to-nose test, objective quantification of reaching accuracy for clinical assessment is still lacking. Emerging VR technology allows for the delivery of rich multisensory environmental stimuli with a high degree of control. Furthermore, recent improvements in the hand-tracking feature offer an opportunity to closely examine the speed, accuracy, and consistency of fine hand movements and proprioceptive function. This study aims to investigate the application of virtual reality (VR) with hand tracking in the rapid quantification of reaching accuracy at the bedside for patients with cerebellar stroke (CS).

**Methods and results:**

Thirty individuals (10 CS patients and 20 age-matched neurologically healthy controls) performed a simple task that allowed us to measure reaching accuracy using a VR headset (Oculus Quest 2). During this task, the participant was asked to reach for a target placed along a horizontal sixty-degree arc. Once the fingertip passed through the arc, the target immediately extinguished. 50% of the trials displayed a visible, real-time rendering of the hand as the participant reached for the target (visible hand condition), while the remaining 50% only showed the target being extinguished (invisible hand condition). The invisible hand condition isolates proprioception-guided movements by removing the visibility of the participant’s hand. Reaching error was calculated as the difference in degrees between the location of the target, and where the fingertip contacted the arc. Both CS patients and age-matched controls displayed higher average reaching error and took longer to perform a reaching motion in the invisible hand condition than in the visible hand condition. Reaching error was higher in CS than in controls in the invisible hand condition but not in the visible hand condition. Average time taken to perform each trial was higher in CS than in controls in the invisible hand conditions but not in the visible hand condition.

**Conclusions:**

Reaching accuracy assessed by VR offers a non-invasive and rapid approach to quantifying fine motor functions in clinical settings. Furthermore, this technology enhances our understanding of proprioceptive function in patients with visuomotor disabilities by allowing the isolation of proprioception from vision. Future studies with larger cohorts and longitudinal designs will examine the quantitative changes in reaching accuracy after stroke and explore the long-term benefits of VR in functional recovery.

## Background

The cerebellum is a complex structure that plays a crucial role in sensorimotor integration and coordination of fine-tuned movements [1]. Proprioception, the internal sense of limb and body position, involves the processing and integration of various sensory inputs from muscle, joints, and tendons by the cerebellum to guide motor movements. Cerebellar dysfunctions have a major impact on the quality of life due their impact on the ability to perform many activities of daily living [2]. There are many causes of damage to the cerebellum including lesions from ischemia, trauma and toxins, tumors, autoimmune and neurogenerative conditions. A classic sign of damage to the lateral cerebellum is dysmetria, the inability to correctly measure distance in body movements. The high prevalence of dysmetria in patients with cerebellar stroke makes these patients ideal candidates to study reaching performance as an indicator of cerebellar function.

Dysmetria in cerebellar ischemic injury can be quickly detected in a neurological examination with the finger-to-nose test. The clinician raises a finger in front of the patient and asks him to touch it with his finger, then touch his nose, and repeat that sequence several times. This simple task tests the patient's ability to pinpoint and reach the spatial position of a target. Cerebellar pathology can lead to overshooting (hypermetria) or undershooting (hypometria) of the reaching motion [3, 4]. There are currently no specific treatments for dysmetria, and the inability to successfully produce fine motor movements significantly affects the quality of daily living and can have major impacts on quality of life and mental health [5–8]. Assessment of severity of cerebellar damage is still dependent on expert clinical experience and likely subjective in nature making it difficult to track change over time [9]. An objective assessment of dysmetria such as a quantification of reaching accuracy would be very valuable in monitoring performance changes in clinical progression or during rehabilitation.

The immense potential of Virtual Reality (VR) is its multidimensional adaptability, as it allows for the delivery of rich multisensory environmental stimuli with a high degree of control [10]. The environments are constructed to be responsive to the user’s input, encouraging the subjects to behave authentically like how they would in a real-world situation instead of in a traditional lab setting [11]. VR with concurrent hand-tracking offers the potential to objectively measure dysmetria in the clinical setting. In the past decade, VR research has received a great deal of attention, specifically in assessing motor competencies and visual perception following recent strokes [10]. Built-in hand-tracking technology such as the Oculus Quest (Meta, USA) allows for high-fidelity tracking of specific parts of the hand in real time, enabling a more accurate assessment of spatial awareness and coordination of fine motor skills [12]*.* Abdlkarim et al. (2024) demonstrated that the Oculus Quest 2 provides reliable data comparable to high-precision systems such as Vicon and OptiTrack, which utilize multiple cameras and reflective markers for detailed and accurate movement data. The study emphasized the Quest 2's validity in clinical settings, although it noted some limitations, such as limited accuracy in capturing fine motor movements and environmental sensitivity. Specifically, the paper reports an average fingertip positional error of 1.1cm, an average finger joint angle error of 9.6^∘^ and an average temporal delay of 45.0 ms [13]. A recent study by our group demonstrated the feasibility of using this technology to measure reaching accuracy in healthy young adults and two individuals with recent cerebellar strokes. Importantly, we were able to uncouple proprioception and vision by manipulating the visibility of the digital rendering of the hand [14]. Teasing apart different sensory modalities was historically a challenge in action-perception research [15, 16]. Looking at proprioception in isolation from vision will provide a clearer understanding of the proprioceptive impairment in cerebellar stroke as well as how visual feedback can potentially provide a compensatory pathway that can mask the true degree of impairment.

In this study, we extend upon our previous work to examine the speed, accuracy, and consistency of fine motor movements of the hand in a larger cohort of individuals with recent cerebellar stroke as well as an age-matched control group. By further investigating how cerebellar stroke affects reaching ability when proprioception is isolated, we hope to better understand how vision and proprioception interact and contribute to dysmetria. Accurate, impartial, and replicable quantification of dysmetria may also be used as a biomarker to track progression or improvement and allow for the development of therapies that can assist with recovery.

The goal of this study was to use VR to compare reaching accuracy and speed between a visual-proprioceptive integration condition and an isolated proprioception condition in older adults who had suffered a cerebellar stroke in the past 6 months, as well as in age-matched neurologically healthy adults. A secondary goal was to perform a preliminary investigation into the feasibility and tolerability of using VR evaluations in subjects with recent stroke, in order help guide future study designs in this patient population.

## Materials and methods

### Recruitment

All participants involved in the study were recruited from the University of Rochester Medical Center (URMC) and the greater Rochester community. Potential subjects for recruitment were identified using the UR CTSI Participant Registry, STUDY00001978. Subjects with cerebellar stroke were recruited from the inpatient stroke service and the outpatient stroke clinic at the Comprehensive Stroke Center. One patient described in Isenstein et al 2022 (patient 1) was included in the analysis. Every participant completed a demographic survey as well as written informed consent as approved by the University of Rochester Research Subjects Review Board (STUDY00003874). Participants demographics are detailed in Table 1.

**Table 1:** Demographic characteristics of all study subjects

	**Controls**	**Patients**
**Number of Subjects**	20	10
**Gender (F/M)**	8/12	1/9
**Age**	66.9 (6.61)	64.7 (9.14)
**Dominant Hand (R/L)**	17/3	8/2
**Race (White/Black/Asian)**	18/1/1	9/1/0
**Type of Stroke** **(Bilateral/Right/Left Cerebellar)**		4/3/3

Age represents mean (standard deviation)

Inclusion criteria for the cerebellar stroke patients included: (1) imaging-confirmed cerebellar stroke (either ischemic or hemorrhagic), (2) sufficient upper extremity strength such that the participant can lift both upper extremities antigravity and reach toward a target, and (3) age 18 years or older (no maximum age cut-off). Exclusion Criteria included: (1) inability to participate in occupational or physical therapy, as determined by their physician, (2) prior history of arm or visual impairments, (3) language or cognitive impairments such that the subject cannot follow simple instructions and complete reach-to-target task.

Of note, while no participants of the current study had any visual impairments, patient 1 described in Isenstein et al. 2022 had horizontal diplopia. However this minor impairment did not affect their ability to complete the VR tasks thanks to the customizable inter-pupillary distance of the VR headset.

### Equipment

The VR experiments were conducted using a Meta Quest 2 headset equipped with four infrared cameras on the exterior which are used by the headset to identify and track objects around the wearer. Using built-in proprietary computer vision algorithms, the Meta Quest 2 identifies the landmarks of the hand including the fingertips and the joints to estimate hand pose and finger angles in real time [17]. Our previously described VR task makes use of this built-in hand-tracking system to measure hand-reaching accuracy and timing during a virtual reality task [14].

### Virtual Reality Task

Upon entering the VR environment, participants were presented a gray, featureless background. Visual renderings of the participants’ hands, which articulated real-time movements of their actual hands, were displayed. Participants viewed instructions which were displayed directly in front of them and were asked to verbally repeat the instructions. Each individual reaching distance was registered by having the participant raise their arm to shoulder height (shoulder flexion) then fully extend the arm and index finger straight forward. A red beam originating from the center of the headset extending forward is used as a guide. The measurement from the tip of the extended index finger to the center of the headset was set as the radius for the placement of the target stimuli during the task to account for individual variability in fully extended reaching distance.

Once the task began, a pink sphere with a 2.5cm diameter positioned along a 60-degree arc at the distance described above would appear in front of the participant. Participants were instructed to reach for this target using the index finger of their left hand. As soon as the fingertip either crossed the center of the target or extended beyond the radius of the arc, the target immediately disappeared regardless of whether the fingertip made contact with it. Subsequently, participants were guided to touch a green cube with 2.5cm x 2.5cm x 2.5cm dimensions, positioned just in front of their chest. This cube served as a reset point that would present once the target sphere vanished. After the cube is touched it would vanish and a new target sphere would randomly appear along the 60-degree arc after a 500ms delay. Participants performed some practice trials until they felt comfortable with the movements and the experimenter determined their readiness to commence the actual task.

In this study, the VR task consisted of two blocks, each involving the participant reaching for the pink target spheres for 10 practice trials and 200 experimental trials. All participants performed the first block with the left hand and the second block with the right hand, except for patient 1 described in Isenstein et al. 2022 who only completed the task with their right hand.

The experimental trials were arranged in a random order to ensure that half of the time a visible rendering of the hand was displayed as the participant reached for the target (referred to as the “visible hand condition”). The remaining half did not display a rendering of the hand and only showed the target vanishing once the finger crossed the target or passed beyond the arc (referred to as the “invisible hand condition”). In the invisible hand condition, participants were deprived of the visual cues to guide their reaching movements and had to rely solely on proprioception [14].

The VR task has been described in Isenstein et al. 2022 [14] in the Visible/Invisible Hand experiment. For specific details as well as a visual demonstration of the VR task please refer to the original study.

### Experimental setup

To monitor participants’ experiences as they engaged with the VR environment, researchers employed SideQuest, a third-party application, in combination with the scrcpy plugin. During the VR sessions, participants were seated on stationary chairs. The settings varied depending whether the participants were inpatient, outpatient, or controls. In the outpatient and laboratory settings, experiments took place in designated rooms, while in the inpatient settings, participants were seated next to their hospital beds.

Participants were given specific instructions regarding their posture and interaction with the VR equipment. They were instructed to maintain contact between their shoulders and the backrest of the chair for the duration of the VR task. Additionally, the Oculus Guardian system, which typically alerts users when they approach the boundaries of a designated safe area, was disabled to prevent any disruptions during the experiment.

Participants were also guided on how to wear the headset properly for comfort and secure placement during the activity. They were instructed to adjust the straps for a snug fit and to customize the inter-pupillary distance slider located at the bottom of the headset.

All experiment environments were carefully set up to optimize conditions for the participants. This included ensuring good lighting, minimizing ambient distractions, and removing any surrounding objects that could potentially impede arm movements during the tasks. Two researchers were present in the room to provide continuous monitoring and occasional reminders as needed to ensure safety and compliance with the instructions. Patients were monitored for any side effects during the study, and reminded that they could end the study at any time should they choose.

### Post-stroke motor functions assessment

To investigate any relationship between reaching performance by VR and well-established functional measures in stroke, all participants underwent an assessment of upper and lower extremity motor functions with a scoring system based on an abbreviated version of the Fugl-Meyer (FM) Motor Scale and the dysmetria component of the Brief Ataxia Rating Scale (BARS) [18, 19].

### Statistical analysis

The reaching accuracy was determined by reaching error, which is the difference in degrees between the center of the target sphere and where the tip of the index finger passed through any point along the 60-degree arc where the target could appear. The cost of removing vision was calculated by subtracting the average reaching error in the visible hand condition from the average reaching error in the invisible hand condition. The reaching time was defined as the amount of time between when the target appeared and when the participant’s index finger crossed the arc. Details about reaching error and reaching time calculation have been previously described in Isenstein et al. 2022 [14].

Reaching time outliers, values which were 3 standard deviations away from each subject’s average reaching time, were removed from the analysis. In the control group, 3.40 ± 1.47 outliers were removed from the visible hand condition and 3.00 ± 1.87 outliers were removed from the invisible hand condition. For the patients, a mean of 2.80 ± 1.87 outliers were removed from the visible hand condition and 2.60 ± 1.51 outliers were removed from the invisible hand condition. Independent samples t-test revealed no difference between the control group and patient group in the number of outliers excluded.

Slope of reaching error was determined as the slope of the best-fit line of 50 or 100 reaching errors over 50 or 100 trials in the VR task. One sample t-test was conducted to assess whether the mean slope of reaching error of all participants in a certain group differed from zero.

Statistical testing was done with SPSS software version 28 (IBM Corp, Armonk, NY, USA) or MATLAB 2021a software (Mathworks, Natick, MA, USA) and figures were generated with GraphPad Prism version 9 (GraphPad Software, San Diego, CA, USA). Shapiro-Wilk tests of normality were conducted on reaching time and accuracy in each condition in all experiments, with one or more conditions in each experiment determined as non-normally distributed. Wilcoxon Matched-pair Signed Rank test was conducted to examine within-group differences (e.g. Control Invisible Error vs. Control Visible Error). Mann-Whitney test was conducted to examine between-group differences (e.g. Control Invisible Error vs. Patient Invisible Error).

## Results

### Demographics

Thirty individuals were enrolled in the study including ten individuals with cerebellar stroke and twenty age-matched neurologically healthy controls. Patients with cerebellar stroke ranged from 50 to 83 years old (64.7 ± 9.14). The time between the diagnosis of stroke and the administration of the task ranged from 1 day to 135 days. Control subjects ranged from 58 to 74 years old (66.9 ± 6.61). There was no difference in the average age between the two groups (t-test, *p* = 0.509) (Table 1). All control participants contacted through the UR CTSI Participant Registry, STUDY00001978 completed the study. Of the eleven patients with recent stroke who were approached, 10 agreed to enroll in the study. All but one enrolled patients completed the study due to subjective nausea and dizziness. There was no other subjective reporting of nausea, dizziness, headache, eye strain, or any adverse effects during or after the study activities, although 3 subjects reported feeling fatigued after performing the task.

### Controls

**Fig. 1:**
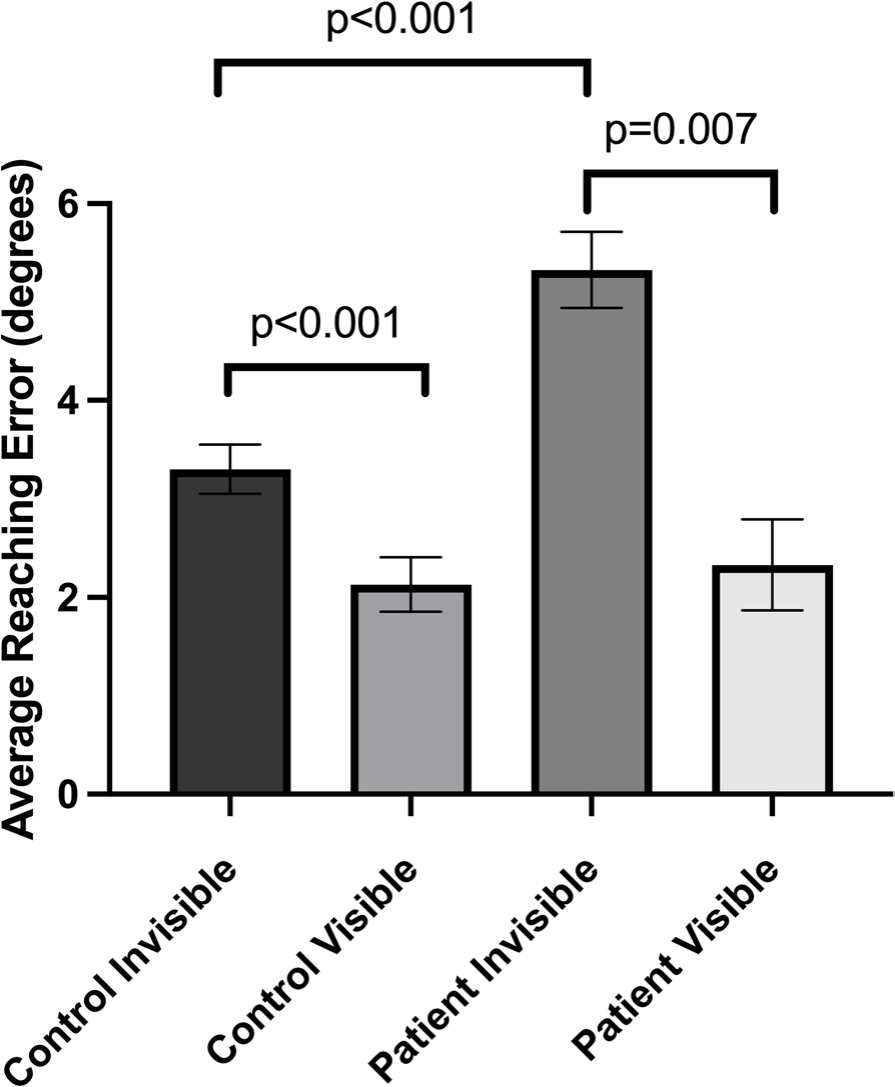
Average reaching error in degrees as a function of hand visibility in control subjects and cerebellar stroke subjects. Error bars denote the standard error of the mean

**Fig. 2:**
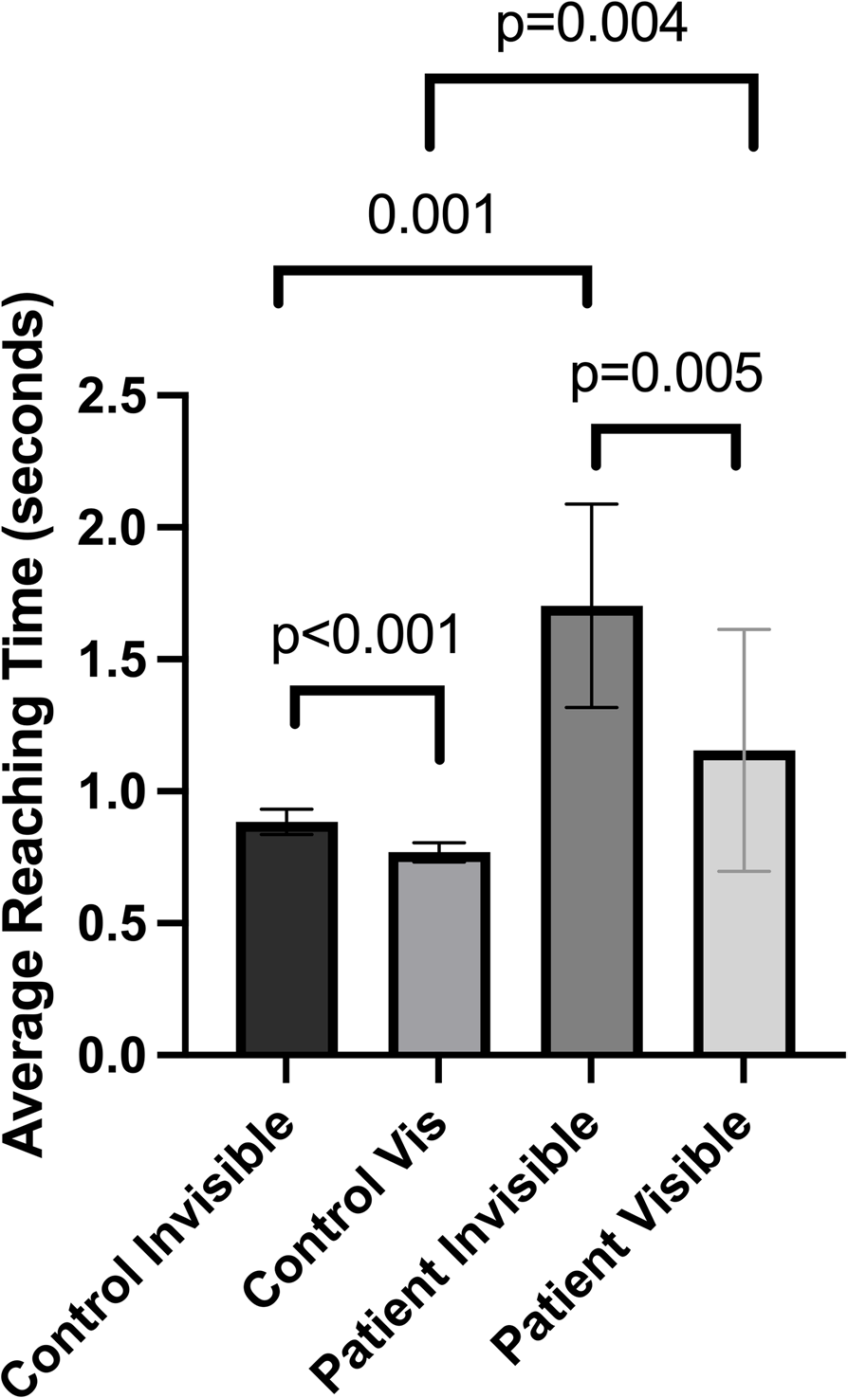
Average time to perform a reaching motion as a function of hand visibility in control subjects and cerebellar stroke subjects. Error bars denote the standard error of the mean

The control participants showed a significantly higher average reaching error in the invisible hand condition (3.29° ± 1.14°) than in the visible hand condition (2.16° ± 1.25°) when both left and right hands are taken into consideration (z = 3.883, *p* < 0.001) (Fig. 1). This result confirms that previous findings in Isenstein et al. 2022 also apply to age-matched neurologically healthy controls. The average reaching time, or the time taken to complete one reaching trial, was significantly higher in the invisible hand condition (0.884s ± 0.215s) than in the visible hand condition (0.769s ± 0.163s) (z = 4.703, *p* < 0.001) (Fig. 2).

### Patients

**Fig. 3:**
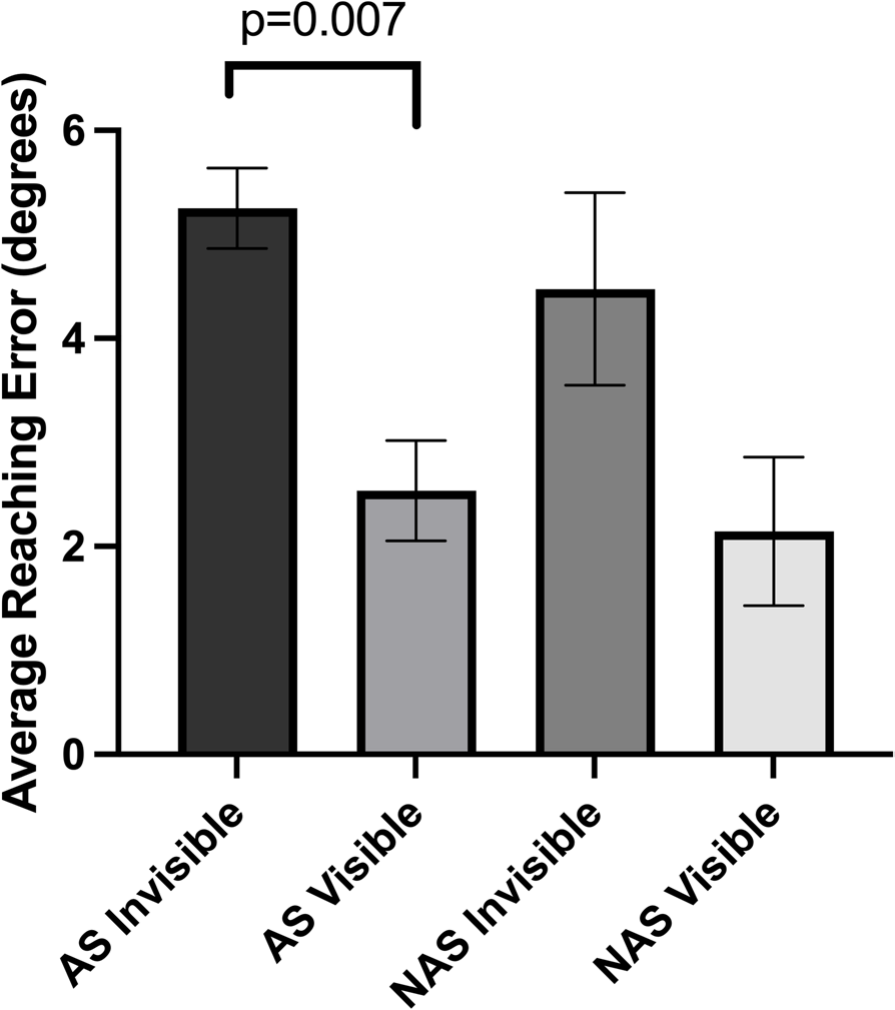
Average reaching error in degrees as a function of hand visibility and hand affected by stroke in cerebellar stroke subjects. Error bars denote the standard error of the mean. *AS* affected side, *NAS* nonaffected side

Like the control group, patient group also showed a higher average reaching error in the invisible hand condition (5.19° ± 1.18 °) compared to the visible hand condition (2.41° ± 1.32°) (z = 2.701, *p* = 0.007) when both hands were included (Fig. 1). When the reaching error was broken down by the side affected by stroke and the side unaffected by stroke, the affected side displayed higher average reaching error in the invisible hand condition (5.25° ± 1.22°) than in the visible hand condition (2.54° ± 1.52°) (z = 2.701, *p* = 0.007), but the unaffected side showed no difference in average reaching error between the invisible hand condition (4.48° ± 2.07°) and the visible hand condition (2.15° ± 1.60°) (z = 1.483, *p* = 0.138) (Fig. 3).

**Fig. 4:**
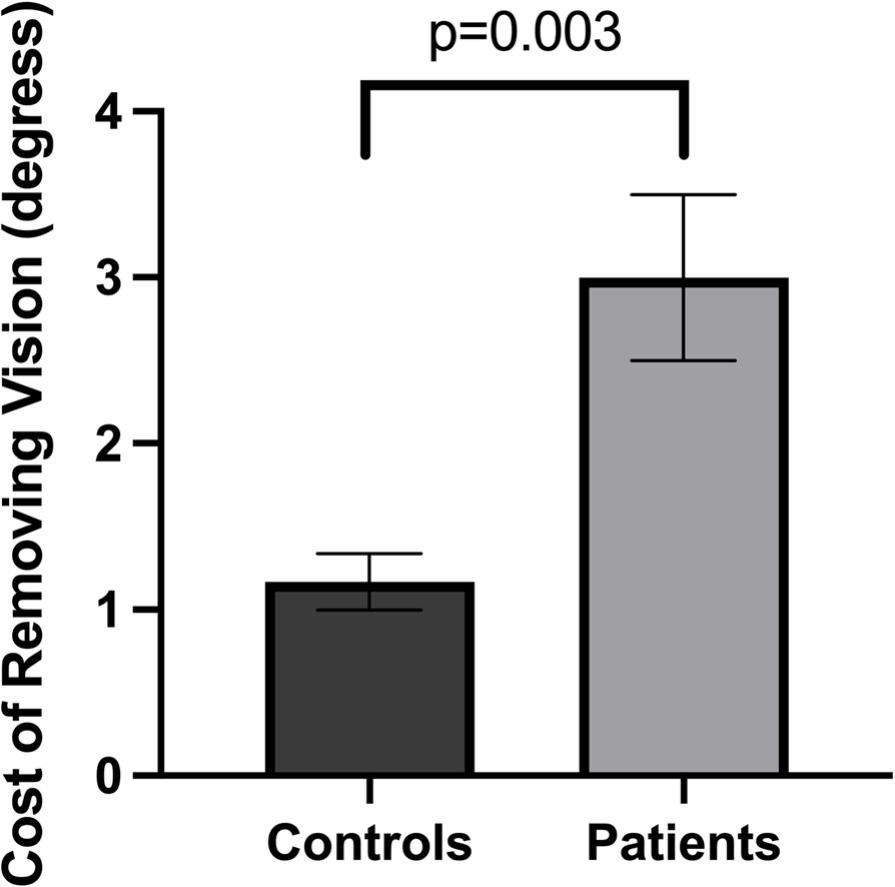
Cost of removing vision in degrees in control subjects and cerebellar stroke subjects. Error bars denote the standard error of the mean

The average reaching time was significantly higher in the invisible hand condition (1.86s ± 1.15s) than in the visible hand condition (1.25s ± 0.58s) when both hands were included (z = 2.803, *p* = 0.005). When the average reaching time was broken down by the side affected by stroke and the side unaffected by stroke, a higher average reaching time in the invisible hand condition compared to the visible hand condition was seen in both the affected side (invisible = 1.74s ± 1.05s, visible = 1.23s ± 0.57s, z = 2.803, *p* = 0.005) and the unaffected side (invisible = 2.20s ± 1.69s, visible = 1.39s ± 0.76s, z = 2.023, *p* = 0.043) (Fig. 4).

All control participants received perfect FM and BARS scores indicating no functional impairments. Therefore the correlation analysis was conducted with our patients group. There was no significant correlations between BARS and average reaching error (*p* = 0.957) or average reaching time (*p* = 0.652).

### Controls vs. patients

The average reaching error of patients with cerebellar stroke (3.80° ± 0.97°) was significantly higher than that of age-matched healthy controls (2.72° ± 1.11°) (U(N_controls_ = 20, N_patients_ = 10) = 30.00, z = 3.080, *p* = 0.001). When the average reaching error was broken down by hand visibility, the average reaching error in the invisible hand condition was higher in patients (5.18° ± 1.18°) than in controls (3.30° ± 1.11°) (U(N_controls_ = 20, N_patients_ = 10) = 17.00, z = 3.652, p < 0.001), but the average reaching error in the visible hand condition was not different between the patients and controls (U(N_controls_ = 20, N_patients_ = 10) = 87.00, z = 0.572, *p* = 0.588) (Fig. 1). The cost of removing vision, defined as the difference in the average reaching error between the invisible hand condition and the visible hand condition, was significantly higher in patients with cerebellar stroke (2.77° ± 1.59°) than in age-matched healthy control (1.17° ± 0.76°) (U(N_controls_ = 20, N_patients_ = 10) = 34.00, z = 2.904, *p* = 0.003) (Fig. 4).

The average reaching time was significantly higher in the patients group (1.60s ± 0.98s) than in the control group (0.85s ± 0.19s) (U(N_controls_ = 20, N_patients_ = 10) = 34.00, z = 2.904, *p* = 0.003). When the average reaching time was broken down by hand visibility, the average reaching time was higher in patients than in controls in both the invisible hand condition (patients = 1.92s ± 1.31s, controls = 0.88 ± 0.22, U(N_controls_ = 20, N_patients_ = 10) = 30.00, z = 3.080, *p* = 0.001) and the visible hand condition (patients = 1.29s ± 0.60s, controls = 0.79 ± 0.17, U(N_controls_ = 20, N_patients_ = 10) = 36.00, z = 2.816, *p* = 0.004) (Fig. 4).

The 10-item Fugl-Meyer motor scale score was significantly higher in controls (100 ± 0.00) than in patients (93.56 ± 7.05) (t = 2.306, *p* = 0.025). The BARS score for ataxia was significantly higher in patients (3.22 ± 2.44) than in controls (0.00 ± 0.00) (t = 2.306, *p* = 0.004). All control participants received respective FM and BARS score indicating no function impairments. One control participant, who deferred FM and BARS testing of the lower extremity due to prior injury but was otherwise neurologically healthy, was excluded from the analysis.

### Change in reaching error over time

To examine the stability of performance and assess for learning or fatigue effects, we estimated the slope of reaching error over the course of the experiments. The control group did not show a slope of reaching error significantly different from zero in either the visible (m = 0.003, SD = 0.03, t = 0.62, *p* = 0.54) or invisible (m = 0.002, SD = 0.02, t = 0.72, *p* = 0.48) hand condition. The control group showed no difference between the slope of the visible hand condition and the slope of the invisible hand condition (*p* = 0.45).

The patient group also did not show a slope of reaching error significantly different from zero in both the visible hand (m = -0.002, SD = 0.02, t = -0.56, *p* = 0.59) and invisible hand condition (m = -0.008, SD = 0.03, t = -1.22, *p* = 0.24). The patient group showed no difference between the slope of the visible hand condition and the slope of the invisible hand condition (*p* = 0.39).

In general, the slope of the reaching error over time is not different from zero in all groups, indicating consistent performance throughout the entire task.

## Discussion

An objective quantification of reaching accuracy is important in evaluating fine motor functions in patients with cerebellar dysfunction. While classic maneuvers such as the finger-to-nose test are fundamental to the bedside detection of dysmetria, they often require clinical expertise, are qualitative and not quantitative, and cannot detect subtle changes over time. VR technology in the recent years has emerged as a non-invasive and efficient way to study action-perception research by allowing researchers a high degree of control over the experimental environment. Furthermore, VR systems such as the Oculus/Meta Quest 2 can provide relatively accurate estimates of hand and finger location and motion [13, 20] and may be potentially adapted to create new methods for measuring movement abnormalities in people with neurological disease.

Our previous work has demonstrated that VR can be used to assess reaching accuracy with and without visual feedback [14]. Two patients completed the VR task and showed the potential for the system to be applicable in clinical populations. This current study expands upon our previous work by including a larger and more diverse patient cohort, thereby demonstrating the feasibility and tolerability of VR in the assessment of reaching accuracy in cerebellar stroke patients with a broader range of clinical deficits and time since stroke. Additionally, direct comparison with age-matched controls offers new insights into proprioceptive impairment in cerebellar stroke. Overall, this study takes a step towards establishing VR-measured reaching accuracy as a potential metric in the evaluation and management of cerebellar stroke patients.

The VR reaching task was successfully administered in a variety of environments including outpatient and inpatient settings. Participants could perform the task either in a laboratory setting or in a hospital room, which was convenient for patients with limited mobility. The task was suitable for patients with a broad range of post-stroke timing (from within the first week to several months after stroke). The total amount of time to conduct a VR reaching task on both arms was approximately 20 minutes. The entire study visit could be conducted by one trained research personnel. There was no report of dizziness, nausea, or vomiting, even though there were some subjective reports of fatigue and that the task felt long. All participants reported no difficulty receiving and following the instructions for the task.

Our results confirmed our previous finding that average reaching error in the neurologically healthy population is higher in the invisible hand condition than in the visible hand condition [14]. One possible explanation for this finding is that relying on proprioception alone, rather than proprioception combined with vision, leads to more reaching error. Uncoupling vision and proprioception also revealed important insights into proprioceptive function in cerebellar stroke. Compared to healthy controls, patients demonstrated higher cost of removing vision and higher average reaching error in the invisible hand condition but not in the visible hand condition. In other words, patients exhibited lower reaching accuracy when depending solely on proprioception but performed similarly to healthy controls when using both proprioception and vision. This finding not only emphasizes the role of the cerebellum in proprioception but also suggests that patients with cerebellar dysfunction are able to compensate, at least for more minor cerebellar deficits, by using visual inputs. Herter et al. 2019 explored the effectiveness of visual compensation in stroke survivors with impaired limb position sense. The study employed an arm-position matching task where stroke patients were asked to observe their limbs during practice. The findings indicated that while some patients could fully compensate for their impaired proprioception using vision, this was not a universal outcome. This suggests that visual input can significantly aid in proprioceptive recovery for some individuals, potentially by reinforcing the brain's representation of limb position through repeated visual feedback. The study also highlights that while visual strategies can be effective, their success may vary among individuals due to differences in the severity of sensory deficits [21]. Newport et al. 2001 further support that vision compensates for proprioceptive function after stroke by providing a mechanistic understanding of how visual inputs can directly enhance somatosensory processing by providing additional sensory cues that help correct proprioceptive errors. Their study on a stroke patient showed that when the patient observed their unseen hand through visual feedback, their felt position of the hand improved [22].

Moreover, patients took more time to complete a reaching motion regardless of hand visibility. Notably, average reaching speed and reaching accuracy appear to be independent from one another in patients with cerebellar stroke. The higher average reaching error in the invisible hand condition in patients suggests that taking more time did not help compensate for the deficit in proprioception. The slower reaching speed could be explained by longer reaching distance to correct for intention tremor, a characteristic feature of cerebellar dysfunction. Other factors such as motivation and fatigue could potentially contribute to these findings. The slope analysis of the change in reaching error over time revealed that the performance of both patients and controls showed no deterioration in over the duration of the task. Future work which examines the reaching distance and quantifies motivation and fatigue will help us better understand how these factors contribute to performance in cerebellar stroke.

Our work contributes to a growing body of literature suggesting that VR be a valuable tool for both the research and clinical purposes. Feitosa et al. (2023) investigated the impact of VR-based rehabilitation on cortical reorganization in stroke patients. The study found significant cortical reorganization in the VR group compared to the control group, suggesting that VR-based rehabilitation can enhance neural plasticity and improve functional recovery. This indicates the potential of VR systems like the Oculus Quest 2 in promoting neural recovery [23]. Takimoto et al. (2021) presented a case where a patient with cerebellar ataxia achieved significant improvement in balance function and returned to work as a standup forklift driver after undergoing VR-guided rehabilitation [24]. A study by Franzò et al. (2023) evaluated a mixed reality system incorporating the Microsoft HoloLens 2 for patients with cerebellar ataxia. Similar to the Oculus Quest 2, the HoloLens is a wearable head-mounted display and has the ability capture kinetics and dynamic measurements of hand movements. While the system helped retrain upper limb coordination, it faced limitations such as lack of accuracy and precision compared to established systems like Vicon and Mocap, and technical challenges in setup and maintenance. The study's findings were based on results from one healthy adult participant, indicating the need for further validation [25]. Our study has taken a step further by applying the Oculus Quest 2 on patients with cerebellar stroke and found that patients were both willing and able to tolerate the VR system.

As compared to existing single camera-based systems such as the Microsoft Kinect [26], there are several benefits of a VR system such as the Meta Quest 2: The VR’s inside-out tracking system, which uses cameras mounted on the headset to track the user’s position in space without the need for external sensors, simplifies the setup and has improved portability. This makes VR systems like the Meta Quest 2 potentially suitable for use in various settings, including home environments, which is a significant advantage for telehealth applications. However, there are also some limitations to consider with VR headsets, including cost and increased sensitivity to lighting and environmental obstacles, which may affect feasibility in home environments.

Our study highlights the unique capability of virtual reality (VR) in decoupling proprioception and vision by manipulating sensory inputs and creating an immersive environment. Valori et al. (2020) demonstrated that VR allows for precise manipulation of visual and proprioceptive cues, enabling the examination of how these sensory inputs integrate in motor control and self-perception [27]. This is particularly important for authentic assessments of cerebellar function and potential quantification of dysmetria. Additionally, Song and Lee (2021) showed that VR-based interventions are as effective as traditional rehabilitation methods for enhancing proprioceptive and motor functions. However, their study focused on functional evaluation and EEG results rather than quantifying performance through VR system outputs [28]. Our study utilized the Meta Quest 2, which features inside-out tracking and hand-tracking capabilities, enabling detailed measurements of hand movements for quantitative analysis. This approach offers a more nuanced understanding of motor functions and proprioception, leveraging the strengths of VR technology in clinical assessments. Reaching accuracy measured in VR could potentially serve as a biomarker to assess the degree of proprioceptive impairment and monitor changes during the recovery process. Furthermore, similar programs could provide therapeutic benefits for individuals whose goal is to practice hand movements using specifically proprioception.

The primary limitations of this study are the relatively small sample size as well as the small distribution of motor impairment severity due to challenges in recruiting patients with high degree of impairment. Even though patients showed a range of stroke chronicity, they all displayed mild levels of motor impairment evidenced by their Fugl-Meyer and BARS scores (Table 2). To improve the generalizability of the findings, future work will aim to include patients with more severe motor impairments due to cerebellar stroke. Additionally, the current system lacks the capability to provide real-time feedback to participants regarding the accuracy of their performance. Participant motivation and fatigue could potentially impact performance within our current framework. A survey before and after the task could yield subjective insights into whether these factors exert different effects in patients versus controls.

**Table 2:** Clinical characteristics of patients in the stroke cohort

Patient	**Age**	**Days since stroke (at time of recording)**	**Location of stroke lesion**	**Fugl-Meyer**	**BARS**
Patient 1	72	10	Right Cerebellar		
Patient 2	70	6	Bilateral Cerebellar	97.5	6
Patient 3	50	63	Right Cerebellar	100	0
Patient 4	66	3	Left Cerebellar	95.5	1
Patient 5	61	6	Bilateral Cerebellar	78.5	5
Patient 6	57	135	Bilateral Cerebellar	99	5
Patient 7	83	90	Bilateral Cerebellar	90	4
Patient 8	58	78	Left Cerebellar	100	1
Patient 9	65	42	Right Cerebellar	88.5	1
Patient 10	65	10	Left Cerebellar	93	6

Patient 1 is described in Isenstein et al. 2022

The Fugl-Meyer assessment consists of 10 items selected in Lin et al. 2021. Each item is scored 0, 1, or 2 points with higher score indicating higher functionality. All items are assessed on the left side first and then the right side. The final score is calculated using an online artificial network described in Lin et al. 2021. The maximum score is 100 and the minimum score is 0. A higher score indicates higher functionality

The BARS scoring system is adapted from Schmahmann et al. 2009. The maximum score is 30 and the minimum score is 0. A higher score indicates higher degree of ataxia

## Conclusions

In summary, this study demonstrates the feasibility and tolerability of reaching accuracy assessment in patients with cerebellar stroke using commercially available virtual reality headsets. Reaching accuracy assessed by VR offers a non-invasive, rapid, and objective approach to quantifying fine motor functions at the bedside. This technology promises to be useful for both clinicians and researchers alike, offering a valuable tool to evaluate and monitor changes in fine motor and proprioceptive functions in individuals with a range of visuomotor impairments.

## Data Availability

Experimental data used to support the findings of this study are available from the corresponding author upon request.
